# Artificial intelligence – the emperor's new clothes?

**DOI:** 10.1177/20552076241287370

**Published:** 2024-09-27

**Authors:** Bjørn Hofmann

**Affiliations:** 1 Centre for Medical Ethics, Faculty of Medicine, University of Oslo, Norway; 2 Institute for the Health Sciences at the Norwegian University of Science and Technology (NTNU) at Gjøvik, Norway

**Keywords:** Artificial intelligence, algorithms, bias, hallucination, hype

## Abstract

There is a massive hype of artificial intelligence (AI) allegedly revolutionizing medicine. However, algorithms have been at the core of medicine for centuries and have been implemented in technologies such as computed tomography and magnetic resonance imaging machines for decades. They have given decision support in electrocardiogram machines without much attention. So, what is new with AI? To withstand the massive hype of AI, we should learn from the little child in H.C. Andersen's fairytale “The emperor's new clothes” revealing the collective figment of the emperor having new clothes. While AI certainly accelerates algorithmic medicine, we must learn from history and avoid implementing AI because it allegedly is new – we must implement it because we can demonstrate that it is useful.

## The emperor's new clothes

Artificial intelligence and machine learning (AI/ML) is expected to transform medicine, generating unprecedented hope and hype^1^ in what has been coined an ‘algorithmic revolution’.^2,3^ However, what is new with AI? Medicine has been an algorithmic endeavour for millennia.

Medical reasoning and decision-making have followed specific (diagnostic, therapeutic, and prognostic) algorithms since antiquity,^4,5^ and medical schools convey algorithmic knowledge to students.^6,7^ Data-driven risk predictive models have been used for decades. Moreover, the vast increase in checklists and guidelines after the emergence of evidence-based medicine has escalated algorithmic medicine substantially.^8,9^ As such, AI/ML just accentuates constitutive characteristics and accelerates an already ongoing tendency.^10^

**Figure 1.**
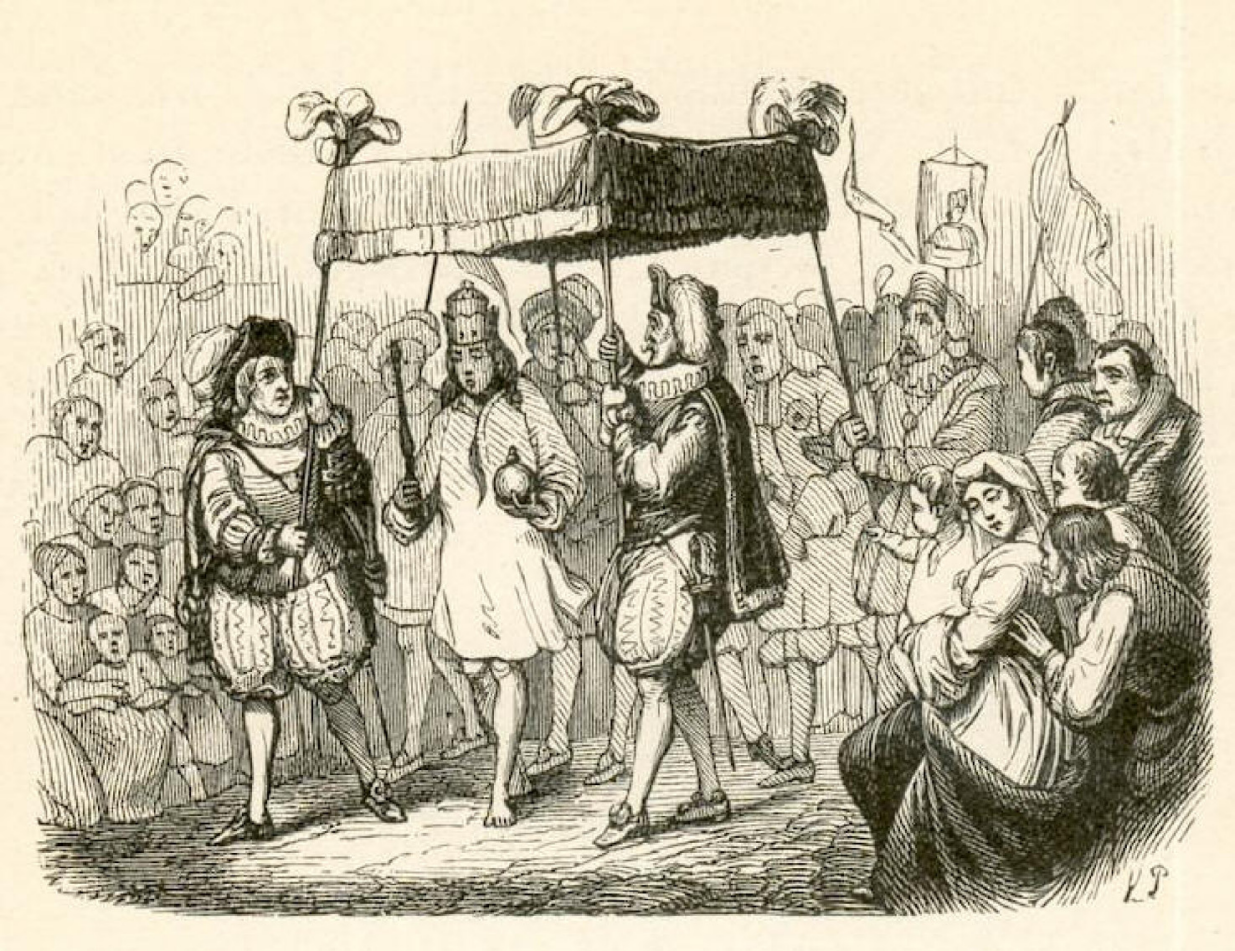
Illustration by Vilhelm Pedersen from the first illustrated version of H.C. Andersen's book *The Emperor's New Clothes* (1837). The image is freely available at https://en.wikipedia.org/wiki/The_Emperor%27s_New_Clothes#/media/File:Emperor_Clothes_01.jpg.

In our euphoric enthusiasm we seem to need somebody like the small child in H.C. Andersen's fairytale about ‘The emperor's new clothes’^11^ to reveal that the Emperor is lured to believe that he has new clothes, but in fact is naked (Figure 1).

Accordingly, we need to expose the hype to address the real challenges of AI/ML. Algorithmic medicine is not new. Neither are the problems with AI/ML, such as hallucinations, biases, transparency (the black box problem), and responsibility evasion.^12–17^

## Algorithmic medicine

The most pervasive use of algorithms in medicine is found in visualizing techniques in radiology and nuclear medicine. Based on targeted ionizing radiation, magnetic fields, ultrasound beams, and radioactive tracers, images are reconstructed by advanced algorithms. The representation of intracorporal structures strongly depends on algorithms that are developed based on studies of many individuals and shaped to obtain and enhance specific clinically relevant characteristics. Thus, algorithms are by no means new.

## New hallucination?

**Figure 2.**
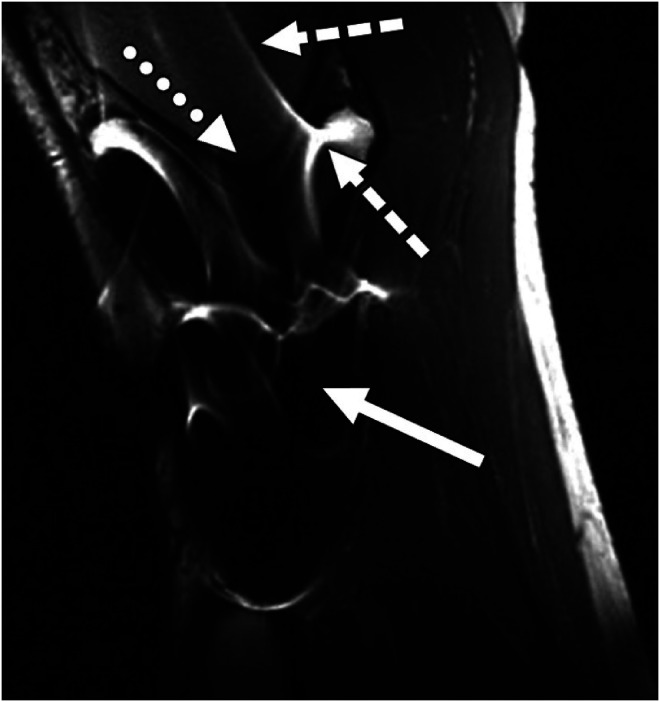
Image artefacts due to the presence of stainless-steel screws in a healthy 37-year-old man. The image is a reprint from Hargreaves et al.^20^ with permission both from the first author and the publisher https://www.ajronline.org/doi/10.2214/AJR.11.7364.

Correspondingly, the algorithms in traditional medicine can generate ‘seemingly realistic sensory experiences that do not correspond to any real-world input. This can include visual, auditory, or other types of hallucinations’.^18^ During the 1920s, medical doctors were able to identify F-cells in the testicles of homosexual men^19^ and more recently metal screws present magnetic resonance imaging (MRI) image artefacts^20^ (see Figure 2).

Clearly, the distortions due to algorithmic image reconstruction have not been classified as hallucinations, but the principle is the same: they provide ‘fictional, erroneous, or unsubstantiated information’.^21^ The history of medicine is full of entities, processes, conditions, and even diseases that in hindsight appear unsubstantiated and unwarranted. Still, researchers defined them, and clinicians diagnosed and treated them. Overdiagnosis is but one example where benign conditions are identified and treated as malign.^22^

While we may be reluctant to think of these ‘perceptual errors’ as hallucinations, the principle is very much the same. The point is that new words for old phenomena do not make the phenomena new.

## Old black boxes

Correspondingly, the so-called black-box problem is not new either. The algorithms for computed tomographies, MRIs, ultrasound, and positron emission tomography/single photon emission computed tomography machines are neither understood by clinicians using them nor by patients benefiting from them. With increasing experience, radiologists have come to trust, appreciate, and apply the algorithmic information, despite occasionally producing false negative and false positive test results.

Moreover, practical breakthroughs have come before causal explanations in medicine for millennia.^23^ The understanding of digitalis came centuries after the acknowledgement of its effects. Uncertainty and unexplainability are not new even when put in black boxes.

## Bias

Likewise, bias has been a problem for medicine in general. Clinicians have been subject to a wide range of biases in diagnostics, prognostics, and treatment,^24–26^ and publication bias has distorted medical evidence production.^27^ Moreover, medical knowledge has been biased in terms of gender, race, age, and other characteristics.^28^ No doubt, AI/ML threatens to enhance this problem, but the problem by no means is new.

## Decision support

Similarly, a wide range of medical decision support systems have been developed, assessed, and implemented long before AI/ML.^29–32^ Many are based on information from large amounts of patients included in previous studies. Moreover, the responsibility for the clinical decisions has been situated with medical doctors using decision support.^33,34^

Although so-called ‘fully autonomous AI systems’ have been approved by the Food and Drug Administration and European Medicines Agency, a wide range of liability and regulatory issues are well-known.^35^ AI/ML-based decision support systems may well accelerate such issues, increasing vendor liability, but the problems with machine-based decision-making are hardly new.

## Alignment and containment

Two other overarching problems have been identified, that is, the problem that AI/ML will incite goals that are not aligned with human goals and values, and the problem that it may become difficult to control AI/ML. While warranted, these challenges are neither new nor unique. Technology has directed medical goals and values, not least through defining medicine's endpoints. Surrogacy endpoints do not always align with hard endpoints.^36–38^ Overdiagnosis and overtreatment are but two examples. Moreover, technology has been difficult to control – in medicine as elsewhere. Hence, AI/ML only pose and potentially enhances general problems with technology.

## What is new? The emperor's new clothes

In sum, algorithms have played a crucial role in medicine since its inception. In the Aphorisms ascribed to Hippocrates of Cos, we learn that ‘[w]hen more food than is proper has been taken, it occasions disease’ and that ‘[a]cute disease come to a crisis in fourteen days’. Clearly, the algorithms of modern medicine have become ever more complex and complicated, not least through precision medicine. However, as argued, the problems with algorithms, such as hallucination, bias, transparency, and accountability are not new.^39,40^ Wrapping uncertainty and unexplainability in black boxes do not render them novel or different.

Clearly, the validation processes are much more challenging,^21^ for example, when algorithms based on enormous amounts of data suggest decisions for (preventing) very rare events, or where the outcomes only can be properly measured in the far future. However, the principles are very much the same as before. Testing hypotheses and validating evidence has always been cumbersome and prone to error.

Certainly, AI/ML is able to analyse vast amounts of data, much faster and in different ways than before. As such, it accentuates and accelerates algorithmic medicine. However, AI/ML-generated algorithms are neither intelligent nor artificial. They are machine-made products that can become very helpful if we harness and govern their development.

Evidently, AI/ML will change the way we do medicine. Professions will change and some professionals be superfluous or find new tasks. However, this is typical for medicine, which has been changed by technology since the invention of the stethoscope by René Laënnec in 1816.

Learning from the little child in Andersen's fairytale and from the history of medicine, we must acknowledge that the introduction of AI/ML does not appear to be radically new. Accordingly, we must reveal the hype^1^ and acknowledge that AI/ML enhances the potentials and perils of medical knowledge production.

We must recognize that new words do not always refer to new things. Instead of being fascinated and misled by a powerful tool, we must harness it so that we can help individual patients in a better and more sustainable way. Even more, we must avoid implementing AI/ML because it is new – we must implement it because of its demonstrated usefulness. Therefore, we must assess AI/ML not on the basis of how new it is, how advanced the algorithms are, and how much data it is trained on, but on how much good it can do for individuals.
